# Evoked craving in high-dose benzodiazepine users

**DOI:** 10.3389/fpsyt.2025.1562622

**Published:** 2025-04-11

**Authors:** Lorenzo Zamboni, Giulia Benvegnù, Francesca Fusina, Roberta Vesentini, Francesca Locatelli, Matteo Mattiello, Vanessa Mannari, Simone Campagnari, Silvia Toldo, Alessio Congiu, Maria Brendolan, Giuseppe Verlato, Cristiano Chiamulera, Fabio Lugoboni

**Affiliations:** ^1^ Unit of Addiction Medicine, Department of Internal Medicine, Integrated University Hospital of Verona, Policlinico “G.B. Rossi”, Verona, Italy; ^2^ Department of Neurosciences, University of Verona, Verona, Italy; ^3^ Department of Diagnostics and Public Health, University of Verona, Verona, Italy; ^4^ Department of General Psychology, University of Padova, Padova, Italy; ^5^ Diagnostics and Public Health-Unit of Epidemiology and Medical Statistics, University of Verona, Verona, Italy; ^6^ Center for Studies and Research in Cognitive Neuroscience, Department of Psychology, University of Bologna, Bologna, Italy

**Keywords:** cue reactivity, benzodiazepine, addiction, virtual reality, abuse

## Abstract

**Introduction:**

Benzodiazepines (BDZs) are among the most abused substances worldwide, and high-dose BDZ abuse is considered a specific type of addiction. Cue reactivity (CR) is a hypersensitivity to motivational stimuli and, in substance use disorders, it increases craving and facilitates relapse, especially in chronic users. Virtual reality (VR) may be a viable technology to implement in CR paradigms. The general objective of this study is the implementation of a VR protocol to identify the causal relationship between the environmental features of a specific setting and craving responses in BDZ abusers.

**Methods:**

Moreover, we investigated the correlation between the degree of BDZ craving and measures of mood, affect, attention, sense of presence, and cybersickness in the subjects, and evaluated the effectiveness that different VR environments have in discriminating between BDZ abusers and controls by comparing the degree of BDZ craving and all of the aforementioned variables in the two groups.

**Results:**

Our data suggest that cues can indeed become conditioned to elicit craving responses in high-dose BDZ abusers, but more studies are necessary to confirm this hypothesis.

**Conclusion:**

Moreover, the use of VR can be a good choice to observe environmental craving for BDZs since it presents a realistic simulation of real-world settings.

## 1 Introduction

Benzodiazepines (BDZs) are positive allosteric modulators of the GABA-A (Gamma-Aminobutyric Acid Type A) receptor (1) and they are widely employed in the treatment of insomnia and anxiety. Despite their widespread use, studies have shown that BDZs should only be prescribed in specific clinical conditions and preferably either intermittently or for short periods of time (2–5). Long-term BDZ use is found in 6% to 76% of total BDZ users (6). It is associated with both adverse effects and dependence, and, therefore, it should only be considered with extreme caution. In particular, it has been associated with cognitive impairments in attention, memory and learning, as well as to a higher risk of incurring in accidents, cognitive decline and delirium (7–16). Moreover, 15 to 44% of long-term BDZ users present moderate-to-severe withdrawal symptoms, and 3–4% exhibit dependence (6).

High-dose (HD) BDZ dependence is considered a specific substance use disorder (SUD) (17), and it has shown to be consistently associated with a lower quality of life (18, 19). A study conducted in Switzerland reports HD BDZ abusers being around 1.5 million in Europe and 600000 in the United States (20), while a cross-sectional survey in France, Germany, Italy, and the UK, reported that approximately 0.14% of the general population took higher-than-recommended doses of anxiolytic drugs (21).

The recommended treatment to reduce BDZs’ impairing withdrawal symptoms comprises either gradual tapering of the dosage or the substitution of the target BDZ with an equivalent dose of another long-acting benzodiazepine, followed by tapering (22, 23).

Concerning the concurrent use of other drugs, it has been noted that BDZs are usually secondary drugs of abuse for most patients. Opioids (54.2%) and alcohol (24.7%) are the two substances that patients most commonly abuse in association with BDZs. Jones et al., in their 2012 review, reported that about 1 in 5 people who abuse alcohol also abuse BDZs.

Craving is one of the most important criteria in the diagnosis of substance use disorders (SUDs; 24) and it is described as a sudden urge to consume a substance of interest (25, 26). This behavior often escalates into compulsively seeking the substance and other conduct related to substance use (27, 28). Indeed, craving is crucial in maintaining abstinence in the long term, and it also has an important impact on other factors, such as the development of the SUD itself, as well as on the course of the treatment (29–31).

Cue reactivity (CR) is a hypersensitivity to motivational stimuli and situations (32). It is considered an adaptive response to environmental cues that represent salient information that the person processes and learns. It can be evaluated psychologically (by assessing changes in mood, craving ratings), physiologically (via skin conductance and heart rate) and behaviorally (gestures/actions) (33). In SUDs, CR is particularly relevant since it increases craving and facilitates relapse. Indeed, subjects that chronically abuse various substances are particularly sensitive to stimuli and situations that have been linked to the pleasurable effects of the target substance (34). In this respect, CR is an evolutionary response which may be both a risk factor, when cues are present, and a protective one, when cues are absent: for example, relapse in smokers has been shown to be reduce in households with no smoking-related cues (35). For this reason, studying the characteristics and the protective or potentially unfavorable valence of various contexts, including external environments, is crucial in the treatment and prevention of abuse-related behaviors. It is also important to use this information in the design of motivationally healthy environments for patients (36). Indeed, the role of domestic and urban settings in inducing goal-directed behaviors is still largely unknown, although the effects of spatial features on affect and perception have been widely examined (37).

Considering this, virtual reality (VR) may be a viable technology to implement in CR paradigms (29, 38, 39). VR simulates multisensory real-life contexts and environments, which are presented in 3D and comprise auditory, visual and/or tactile inputs (40). Compared with traditional CR paradigms, such as photos or 2D images, VR would thus have the advantage of being more ecological, enhancing participants’ *sense of presence*, a state of mind in which virtual environments are perceived as similar to real-world ones. This would increase the validity of this research method compared with traditional ones (41–43).

Furthermore, technical VR features such as immersion within the VR environment, the inclusion of substance-related cues, the presentation of highly realistic settings, and allowing subjects to actively interact with the system through real-time feedback would further enhance this method’s efficacy (44–47).

To the best of our knowledge, there are currently no studies in literature that examine CR and VR in BDZ abuse. Some research groups have investigated CR, smoking and alcohol abuse (45, 48, 49) and have emphasized the influence that environmental settings have on craving in alcoholism and smoking.

This study has several aims: the general objective of the study is the implementation of a VR protocol for the purpose of identifying the causal relationship between environmental features of a specific setting and craving responses in BDZ abusers.

Also, there are two secondary objectives: 1) investigating the correlation between the degree of BDZ craving in the various scenarios and measures of mood, affect, attention, sense of presence, and cybersickness in subjects who abuse BDZs (50); 2) the evaluation of the effectiveness that the three different VR environments have in discriminating between BDZ abusers and control subjects by comparing the degree of BDZ craving and measures of mood, affect, attention, sense of presence, and VR malaise in the control group *vs* those in the experimental group.

## 2 Materials and methods

This research is an experimental study based on the study protocol by Zamboni et al. (51). It aimed to measure the degree of BDZ craving induced by VR exposure to environments containing cues associated with BDZ use after immersion in a VR setting.

The study consisted in a single session lasting approximately 45 mins. Participants filled out the Profile of Mood States (POMS) questionnaire before and after the session as a pre-VR baseline measure concerning their mood and affective state. After a 3-minute VR baseline, we administered three scenarios, each lasting 3 minutes. After the baseline and each of the scenarios, subjects were required to fill out the VAS to report craving, and a modified version of the Alcohol Attention Scale (AAS) questionnaire. At the end of the experimental session, in addition to the POMS, subjects were also asked to fill in the Presence Questionnaire (PQ) to assess their sense of presence, and the Simulator Sickness Questionnaire (SSQ) to assess the presence of possible adverse effects due to VR exposure.

### 2.1 Ethics statement

Approval for the research was obtained from the Ethics Committee for Clinical Trials (CESC) of the Provinces of Verona and Rovigo based at the Integrated University Hospital of Verona, Italy (approval code: 3624CESC with Protocol No. 16883 of 09-03-2022). The latest revision of the Declaration of Helsinki as well as the Oviedo Declaration was the basis for the ethical conduct of the study. The study protocol was designed and conducted to ensure adherence to the principles and procedures of Good Clinical Practice and to comply with Italian law, as described in the following documents and accepted, by signature, by the study investigators: ICH Harmonized Tripartite Guidelines for Good Clinical Practice 1996. Directive 91/507/EEC, The Rules Governing Medicinal Products in the European Community. D. L.vo n. 211 of June 24, 2003. D. L.vo n. 200, November 6 2007. Ministerial Decree of December 21, 2007. AIFA Determination, March 20, 2008. All essential clinical records will be retained to demonstrate the validity of the study and the integrity of the data collected. The promoter of this study, in accordance with the responsibilities required by the rules of good clinical practice (Legislative Decree 211/2003) and in accordance with the laws and regulations regarding data protection (including the European Regulation on the protection of personal data 2016/679), will process the personal data that will be collected exclusively for the implementation of the study and for the purpose of device surveillance.

### 2.2 Participants

The sample was composed by fifty-four native Italian speakers of both sexes (males = 28; age range = 18-65 years), with an average age of 36.89 years (SD ± 11.89).

The following inclusion criteria were applied to the experimental group (N = 27, males = 13): i) BDZ use disorder diagnosis following DSM 5TR criteria (24); ii) being ready to start a treatment at the Addiction Medicine Unit due to the inability to autonomously quit BDZ use; iii) high-dose BDZ abuse (i.e., BDZ intake must be at least five times higher than the defined maximum daily dose -DDD-).

In contrast, the control group (N = 27, males = 15) included subjects without SUDs (including a BDZ use disorder) according to ICD 10 F10-F19.

The following exclusion criteria were applied to both groups: i) having a history of epilepsy or having first-degree family members with epilepsy; ii) having a history of severe cardiovascular or chronic disease; iii) pregnancy; iv) presence of a cardiac pacemaker or other metallic devices on the head and neck, with the exception of piercings and dental braces; v) intake of psychoactive substances which may have interfered with the results of the study.

### 2.3 Instruments & software

**Figure 1:**
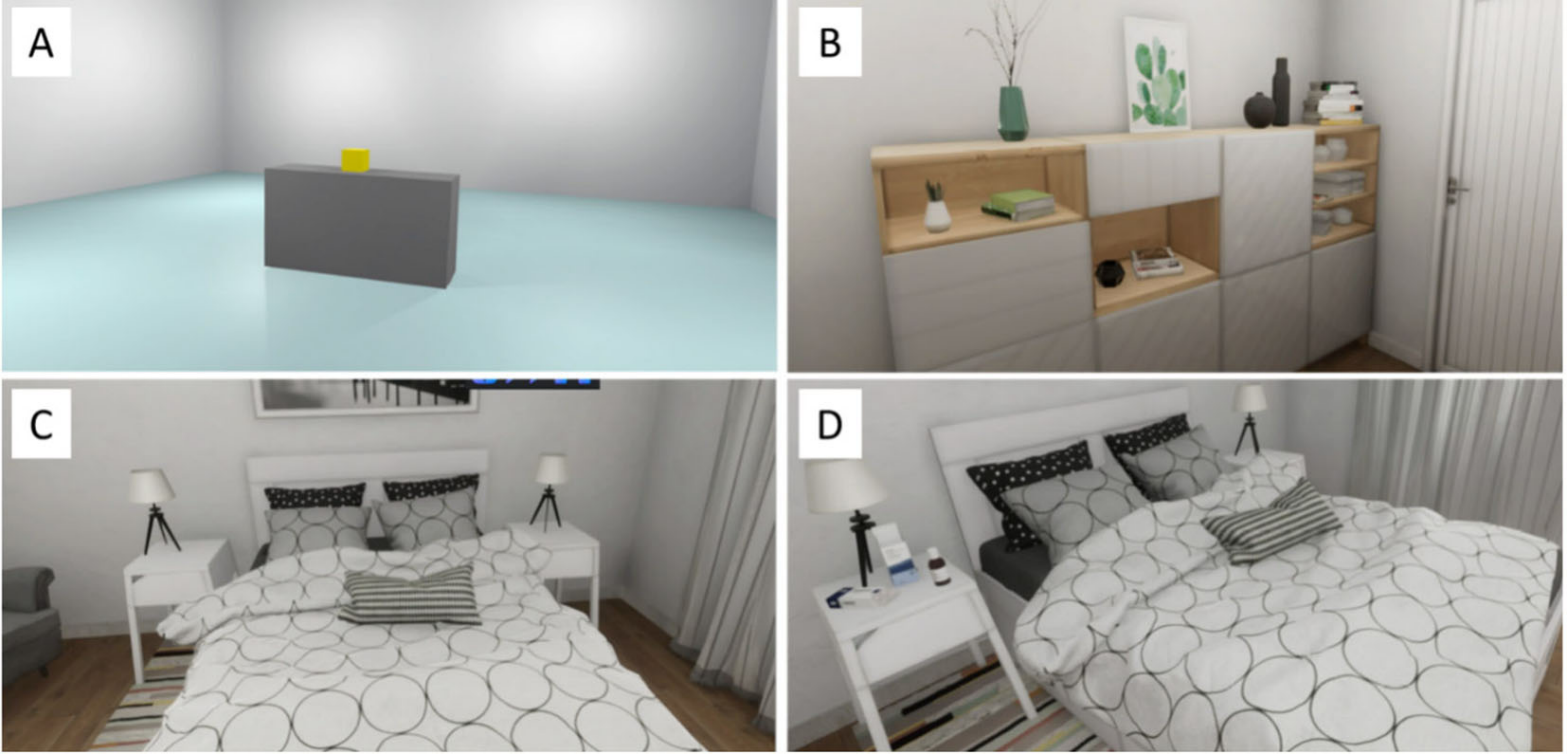
Virtual Reality scenarios. The tutorial scenario **(A)**, the neutral scenario **(B)**, the No Cue scenario **(C)** and the Cue scenario **(D)**.

We used the HTC-Vive VR device, consisting of a Head Mounted Display (HMD), two controllers for interacting with the virtual environment, and two external infrared sensors. All VR scenarios (**Figure 1**) were modelled with the software Blender 2.8 and implemented in Unity 2017.4. Four scenarios were developed: i), a tutorial environment to familiarize participants with VR technology, consisting of a room with a graspable object (a cube). This is the only scenario in which subjects could interact with the experimenter; ii), a neutral scenario representing the entrance of a house. In this scenario, the subject could move freely in the virtual environment, but there were no interactive objects; iii), the No Cue scenario. In this scenario, representing a bedroom, the subject could move freely but no interactive objects were present; and iv), the Cue scenario. This scenario represents the same bedroom as the No Cue scenario but in this case the subject, in addition to moving freely, could interact with BDZ-related graspable objects (i.e., a bottle of Lormetazepam and a box of Alprazolam tablets).

### 2.4 Measures

For a more detailed description of the materials and methods, and in particular of the measures used, see Zamboni et al., (51). The questionnaires employed in the present study are given below.

-*Ad hoc* questionnaire to measure Benzodiazepine (BDZ) craving. The instrument consists of a single-item 10-point scale based on the literature and adapted from Benvegnù et al. (49) and Traylor et al. (48), with a score ranging from 0 (absent) to 9 (extreme).
-*Ad hoc* questionnaire to measure the attention given to BDZ-related cues. The questionnaire is a modified version of the Alcohol Attention Scale (AAS) (52) and it consists of two BDZ-themed questions investigating the attention paid to BDZs (item 1) and the intention to use them (item 2), with an 11-point response scale ranging from 0 to 10.
-Profile of Mood States (POMS). The questionnaire (53) comprises 58 items, with a 5-point scale response, measuring six factors: Tension/Anxiety (T), Depression/Dejection (D), Anger/Hostility (A), Vigor/Activity (V), Fatigue/Inertia (S), and Confusion/Bewilderment (C). The total score is calculated as the sum of the five negative scales (T, D, A, S) minus the positive one (V).
-Simulator Sickness Questionnaire (SSQ). The possible presence of symptoms associated with cybersickness was assessed using the SSQ (54). The questionnaire consists of 16 items, 4-point response scale, and provides a total score and 3 subscales (Nausea, Oculomotor Disturbance, and Disorientation).
-Presence Questionnaire (PQ). The perception of “being present” in the virtual environment was measured using the PQ (55), a questionnaire with a 7-point scale response.

### 2.5 Procedure

**Figure 2:**
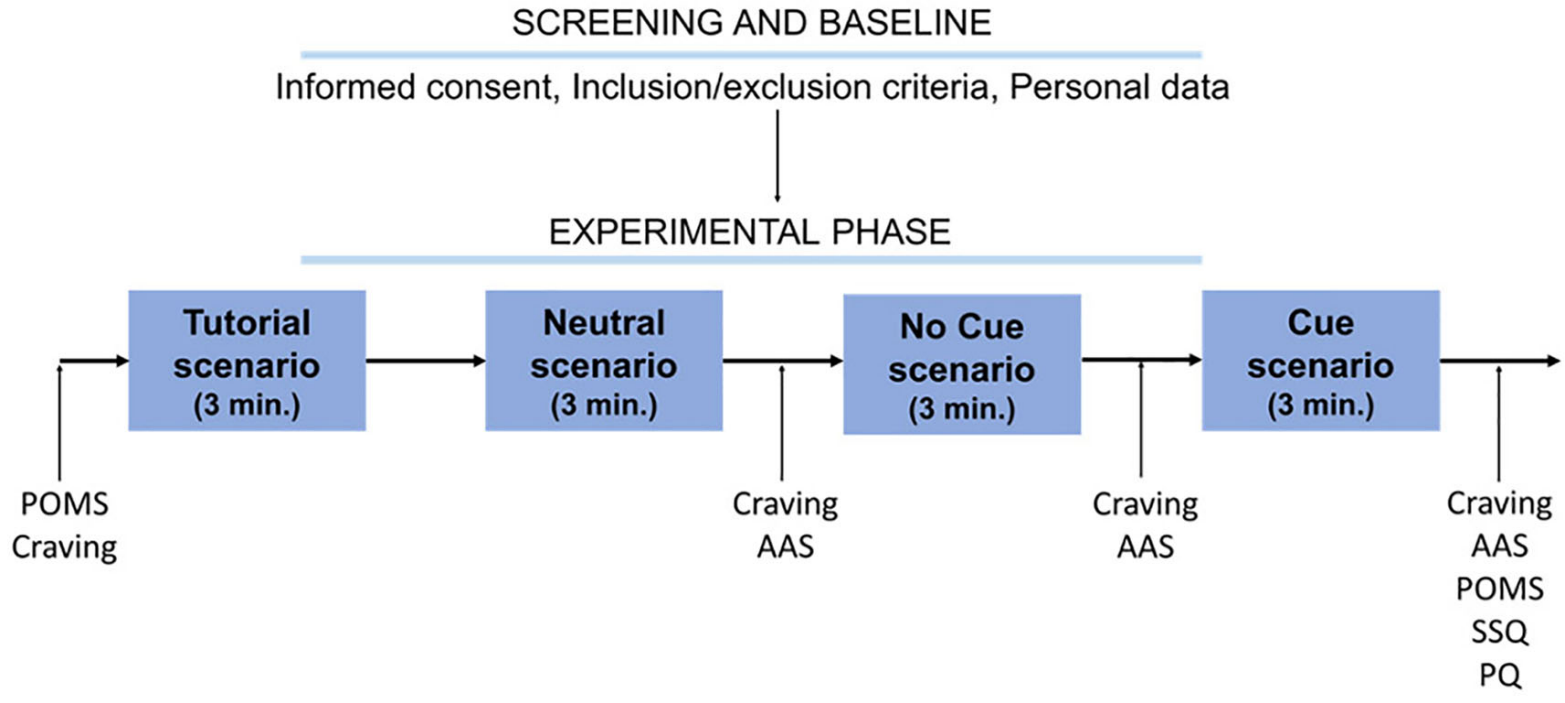
Schematic representation of the procedure. POMS, Profile of Mood States; Craving, craving *ad hoc* questionnaire; AAS, modified Alcohol Attention Scale; SSQ, Simulator Sickness Questionnaire; PQ, Presence Questionnaire.

Each participant was asked to sit in the VR station and received all the necessary information regarding the experiment. After signing the informed consent and verification of eligibility, the subject filled out the questionnaires. Before the VR session began, the POMS and the craving questionnaire were administered (**Figure 2**). The experimenter then instructed the participant on how to use the HTC-VIVE VR device and how to move in the virtual environment. The first scenario was a 3-min baseline simulation (tutorial scenario) during which the subject familiarized with VR and practiced with the device. In a fixed sequence, the other three scenarios were administered, for 3 minutes each: house entrance (neutral scenario), bedroom without BDZs (No Cue scenario), and bedroom with BDZ bottles (Cue scenario). The participant did not have to undertake a specific task but could freely explore the virtual environments by using the joystick and moving their head. At the end of each scenario, the subject removed the visor and headphones and filled out the craving questionnaire and the modified AAS. At the end of the last one, the craving questionnaire, the modified AAS, the POMS, the SSQ, and the PQ were administered. All procedures were approved by the local academic ethical committee (Ethics Committee for Clinical Trials -CESC- of the Provinces of Verona and Rovigo; approval code: 3624CESC with Protocol No. 16883 of 09-03-2022) and followed the principles of the Declaration of Helsinki.

### 2.6 Data analysis

Sample size was estimated by using the G*Power 3.1.5.1 software (56) with an a-priori analysis (α level: 0.017, power level, 1-β: 0.80, effect size f: 0.7. For more details on sample size computation see Zamboni et al., 51).

Since the assumptions for the ANOVA were violated, a Friedman test was performed to test for craving differences between the different timepoints in the experimental group. In case of significance, Dunn’s multiple comparison tests were conducted. The same analysis was also carried out for the scores of the two items in the modified version of the AAS (henceforth, AAS 1 and AAS 2).

To test whether the No Cue and Cue scenarios elicited an effect on the experimental group compared with the control group, a Mann-Whitney series was performed for craving, AAS 1 and AAS 2 scores.

To check for possible associations between craving and the other measures, Spearman correlations were made between the craving, AAS 1 and AAS 2, POMS, SSQ, and PQ scores measured at the same timepoints in the experimental group.

All statistical analyses were conducted using the GraphPad Prism 9.1.0. software (GraphPad, CA, USA).

## 3 Results

**Figure 3:**
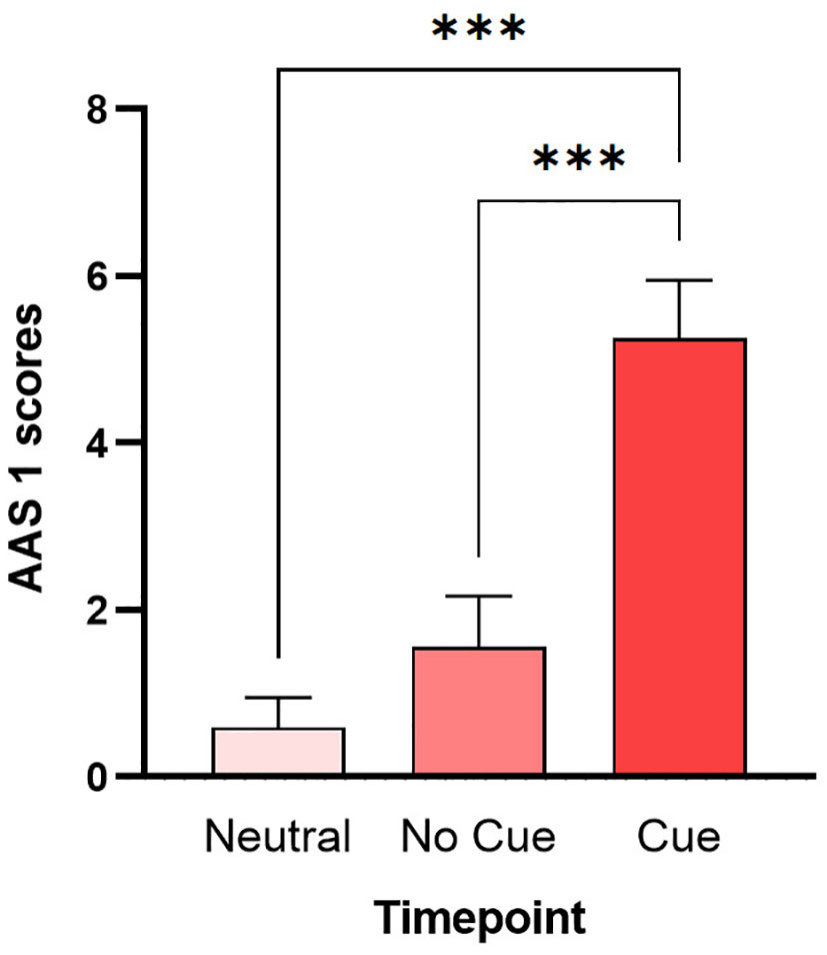
Multiple comparisons of AAS 1. Bars represent the average scores and the S.E.M. of AAS 1 measured after the neutral scenario (Neutral), after the bedroom without BDZs (No Cue), and after the bedroom with BDZ bottles (Cue). *** (p < 0.001) (Dunn’s multiple comparisons test).

The Friedman test on craving scores showed no significant differences between the different timepoints [χ2(3) = 6.45, p = 0.09]. In contrast, the Friedman test on AAS 1 was statistically significant [χ2(2) = 45.23, p < 0.0001]. *Post-hoc* comparisons (**Figure 3**) showed a significant increase in scores in the Cue scenario, both compared to the neutral scenario (p < 0.0001) and compared to the No Cue scenario (p < 0.0001). The Friedman test of AAS 2 showed no significant differences [χ2(2) = 5.09, p = 0.07].

**Figure 4:**
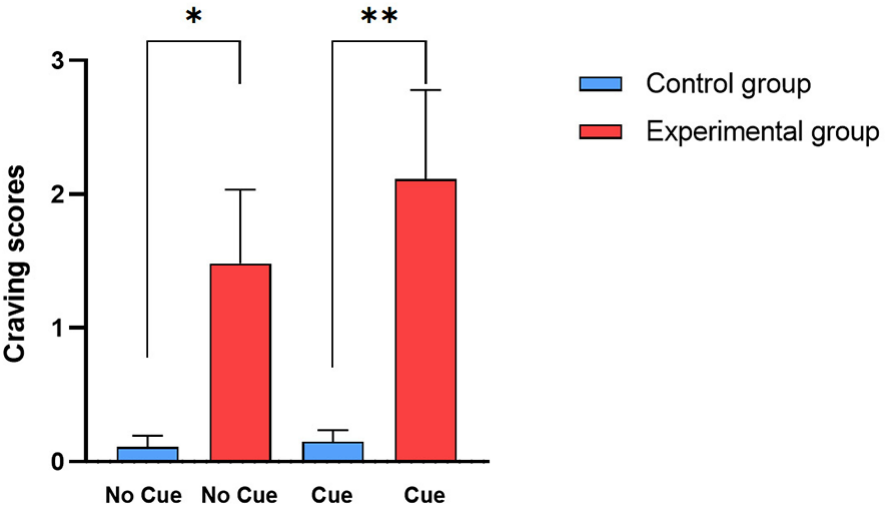
Mann-Whitney of craving scores. Bars represent the average scores and the S.E.M. of craving measured after the bedroom without BDZs (No Cue) and after the bedroom with BDZ bottles (Cue) in the control group (cyan bars) and in the experimental group (red bars). * (p < 0.05) and ** (p < 0.01) (Mann-Whitney test).

The Mann-Whitney on craving scores showed greater craving in the experimental group than in the control group in both the No Cue scenario (U = 290.5, p = 0.031) and the Cue scenario (U = 248, p = 0.006) (**Figure 4**). In contrast, the Mann-Whitneys of AAS 1 and AAS 2 did not reveal significant differences.

**Figure 5:**
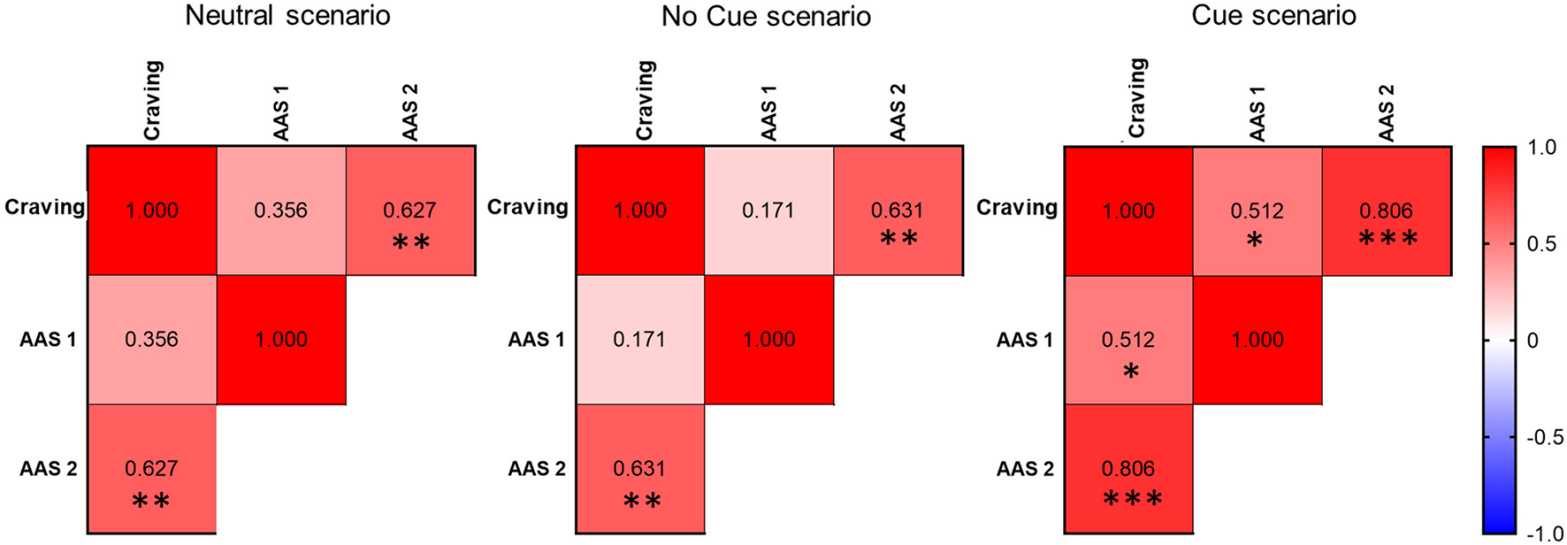
Correlational analysis. Heat map of correlations between craving, AAS 1, and AAS 2 scores measured after the neutral scenario, after the No Cue scenario, and after the Cue scenario. Spearman’s Rho are shown in the cells. * (p < 0.05), ** (p < 0.01) and *** (p < 0.001).

Regarding correlational analyses, craving scores correlated positively with AAS 2 measured after the neutral scenario (rs = 0.62, p = 0.001), after the No Cue scenario (rs = 0.63, p = 0.001) and after the Cue scenario (rs = 0.80, p < 0.0001) and with AAS 1 scores measured after the Cue scenario (rs = 0.51, p = 0.018). AAS 1 and AAS 2 scores measured after the neutral scenario show a positive correlation (rs = 0.58, p = 0.003) (**Figure 5**).

## 4 Discussion

Several papers address BDZ craving, but they address it as a factor concerning abstinence rather than environmental cues (6, 17). There is only a study by McHough et al. (57) that suggests BDZ cues can become conditioned to elicit craving responses and that the degree of cue reactivity is correlated with known risk factors for benzodiazepine misuse. However, this study does not use VR and presents a heterogeneous sample. To overcome these methodological issues, we have included only monoabusers of high-dose BDZs in our study. Furthermore, we used VR to observe if the BDZ cues could elicit a craving response in a sample of BDZ high-dose abusers.

The results show that the experimental group showed an increased attention to BDZ-related stimuli compared to other scenarios in which there were no related cues. This result confirms our expectations and is in agreement with other studies on alcohol and tobacco addiction (58, 59).

Regarding craving, we observed a difference between the control group and the experimental group at the VAS craving scales. The experimental group registered higher craving levels both in no cue and cue scenarios. Similar data was found in studies examining other addictions, particularly tobacco and alcohol (60–63).

Moreover, our study revealed a correlation between craving and attention to BDZs, and between craving and the desire to use BDZ. These data support the hypothesis of cue reactivity in high-dose BDZ abusers. Indeed, the presence of BDZ-related cues in the virtual scenario elicits craving, attention towards BDZs and the desire to use these drugs.

Our study confirms the feasibility of VR as a research method because no subjects withdrew from the experiment due to motion sickness or other issues related to VR simulation; this data is therefore encouraging concerning the use of VR to observe behaviors related to BDZ addiction.

This is the first study analyzing CR in the high-dose BDZ abuser population, revealing a correlation between these two aspects. The data is significant as it has never been collected before. Additionally, this aspect may pave the way for new future therapeutic scenarios, placing greater emphasis on the lifestyle context of this type of patients. Just like with other substance addictions (alcohol, smoking, etc.), intervening in such a context could lead to a reduction in the number of relapses.

In addition, the use of VR in the treatment of high dose BDZ abuser could become a valuable clinical tool. However, as emphasized in the review written by Wiebe et al. (64), the times are not yet ripe to strongly assert the effectiveness of this methodology. In this regard, further studies will be needed.

Our study presents several limitations: we measured craving using only a VAS scale; also, the sample is small, and although we have respected the initial power analysis results, nevertheless a large sample size could strengthen our results, finally, we did not collect certain sociodemographic information (time of onset of BDZ use disorder, the lifetime use of other substances, or a family history of SUD and other psychiatric diseases) that could have enriched the analysis. Another potential bias could be the mean age difference between two groups, and also the fact that the patients’ BDZ intake data were missing. This aspect could increase data specificity, since, in our study, the BDZ dosage was only self-reported by the subjects.

## 5 Conclusion

Our data suggest that cues can indeed become conditioned to elicit craving responses in BDZ high-dose abusers, but more studies are necessary to confirm this hypothesis. Moreover, the use of VR can be a good choice to observe environmental craving for BDZs since it presents a realistic simulation. Future studies could measure craving by using more objective measures (EEG, heart rate, skin conductance etc.), and not only in high-dose abusers but also in long-term abusers.

## Data Availability

The raw data supporting the conclusions of this article will be made available by the authors, without undue reservation.
